# Mapping Obesity Care Pathways and Healthcare Resource Use From 2015 to 2019 in England

**DOI:** 10.1002/osp4.70173

**Published:** 2026-07-19

**Authors:** Alexander Dimitri Miras, Silvia Capucci, Sylwia Migas, Bozydar Wrona, Liwei Zhao, Johann Castañeda‐Sanabria, Lise M. Hagelund, Vasileios Antavalis, Camilla S. Morgen

**Affiliations:** ^1^ Imperial College London Hammersmith Hospital London UK; ^2^ School of Medicine Ulster University Derry UK; ^3^ Novo Nordisk A/S Søborg Denmark; ^4^ NorthWest EHealth Ltd Manchester UK; ^5^ IQVIA Mölndal Sweden; ^6^ IQVIA London UK; ^7^ Novo Nordisk Copenhagen Denmark; ^8^ Novo Nordisk Gatwick UK

**Keywords:** database research, health economics, metabolic and bariatric surgery, obesity care, observational study, weight management

## Abstract

**Objective:**

This observational study characterized obesity care pathways and healthcare resource use in England between 2015 and 2019.

**Methods:**

Data were obtained from Discover and the Salford Integrated Record (SIR), two databases of linked primary and secondary electronic health records in England. Adults with a body mass index (BMI) ≥ 30 kg/m^2^ who attended obesity clinics were included over 4 years (January 1, 2015 [SIR] and January 1, 2016 [Discover] to December 31, 2018).

**Results:**

Overall, 1698 people living with obesity were included from Discover and 561 from SIR. Most (74.9%–78.6%) received a lifestyle intervention as their first intervention, whereas 6.2%–16.3% received a pharmacological treatment or metabolic/bariatric surgical procedure. Time to first intervention was typically > 6 months, and most individuals remained in their baseline BMI group after 8 months' follow‐up. Obesity‐related complications resulted in high annual per‐person direct healthcare costs, particularly for acute cardiovascular events such as myocardial infarction (Discover: 2285 GBP; SIR: 2194 GBP) and incident stroke (Discover: 3005 GBP; SIR: 1550 GBP).

**Conclusions:**

In conclusion, the time to first intervention can be considerable for people living with obesity in England and these individuals may incur high healthcare costs. There is an unmet need for timely and effective weight management in England.

**Abbreviations**
BMI: body mass index
EHR: electronic health record
GBP: British pound sterling
HCRU: healthcare resource utilization
IQR: interquartile range
NHS: National Health Service
ORC: obesity‐related complication
SD: standard deviation
SIR: Salford Integrated Record
T2D: type 2 diabetes

## 1 Introduction

Early intervention and treatment are important goals for the management of obesity and addressing obesity‐related complications (ORCs) [1, 2]. In the UK, the Department of Health Working Group first proposed a four‐tier framework for obesity management services in 2014. Typically, Tier 1 focuses on universal interventions aimed at prevention, and Tier 2 includes lifestyle and obesity management services, with the aim of supporting diet and behavioral changes. Tier 3 consists of clinician‐led obesity management services delivered by a multidisciplinary team. Tier 4 offers specialized services for complex obesity cases, including metabolic and bariatric surgery [3, 4]. However, the availability of specialized weight management services varies across the UK [5], meaning that eligible individuals in some areas are unable to access support and treatment. A report published in late 2024 indicated that less than 60% of Integrated Care Boards in England (24 out of 42) commissioned both Tier 3 and Tier 4 services, and nearly 20% of local health areas lacked a metabolic and bariatric surgery service [6]. A UK survey of people living with obesity has also highlighted long delays between their noticing difficulties with weight and discussing weight loss with a healthcare professional, as well as a low rate of recommended referrals to specialist obesity treatment services [7].

Studying treatment use and care pathways in obesity is essential for understanding access to care, treatment decision‐making, and the effectiveness of interventions, and to identify which groups of people benefit most from treatment. Published data indicate that many individuals with obesity in the UK do not receive any intervention, and only a minority receive pharmacological or surgical intervention. A retrospective study of individuals diagnosed with obesity in primary care in the UK between 2010 and 2019 reported that approximately half of individuals (52.6%) received lifestyle support in the year before or after initial diagnosis and only 8.2% received medication to treat obesity [8]. Another UK study, focusing on primary care between 2005 and 2012, reported that only 10% of individuals with body mass index (BMI) 25–30 kg/m^2^ and 41% of individuals with a BMI ≥ 40 kg/m^2^ received a weight management intervention [9]. A review of metabolic and bariatric surgery in the UK has reported that NHS procedures address less than 1% of the need and that although the UK has the second highest rate of obesity in Europe, it ranks 13th out of 17 countries for rates of metabolic and bariatric surgery [10]. In a previous study of the English primary care population (2007–2020), 1.1% of individuals with BMI > 40 kg/m^2^ or BMI > 35 kg/m^2^ with complications received metabolic/bariatric surgery [11], and in a study covering the period 2010–2019 using data from linked primary and secondary electronic health records, only 2.4% of a population eligible for metabolic/bariatric surgery ultimately received surgery [12].

Data from other European countries also indicate relatively low rates of intervention for obesity management. The RESOURCE survey collected data on healthcare resource utilization (HCRU) and weight loss strategies for individuals with a self‐reported BMI ≥ 30 kg/m^2^ across France, Germany, Italy, Spain, Sweden, and the UK, between 2020 and 2021 [13]. Reported rates of metabolic and bariatric surgery were 1.5%, and 12.3% of respondents received a prescription medication for weight management. The majority of respondents who answered questions regarding estimated weight changes (73.4%) had not experienced weight loss of 5% or more in the past year [13].

Despite the availability of data regarding obesity management in the wider UK, and the known limitations in access to specialist obesity support in England, there is limited real‐world evidence on individual care pathways and experiences with obesity management services in clinical practice in England, the effectiveness of the obesity care pathway or the associated HCRU, including the impact of treating ORCs [3]. The present observational study sought to address this evidence gap using data from two obesity treatment centers/clinics to assess the real‐world obesity care pathway in England between 2015 and 2019. The study focused on the period before the availability of glucagon‐like peptide‐1 receptor agonists for the treatment of obesity in the UK [5, 14], to provide insights into the obesity landscape before these medications became available. This analysis described the characteristics of people living with obesity in England being treated in obesity specialist care; explored their interactions with the healthcare system and healthcare professionals; summarized the obesity treatment received; and assessed the HCRU and direct costs associated with treating ORCs.

## 2 Materials and Methods

### 2.1 Data Sources

Data were obtained from two electronic health record (EHR) databases in England: Discover and the Salford Integrated Record (SIR). Discover is a real‐world database of linked primary and secondary EHRs covering 2.7 million people residing in North West London [15, 16]. Primary care data are available from January 1, 2010 onwards, with linkage to secondary care data available from January 1, 2015. The SIR contains comprehensive primary and secondary EHRs for nearly all patients in the Salford region in Greater Manchester, North West England, starting from 2009 [17]. Data from primary and secondary care in the Salford region were linked from January 1, 2010 to December 31, 2019.

### 2.2 Study Design and Population

**FIGURE 1:**
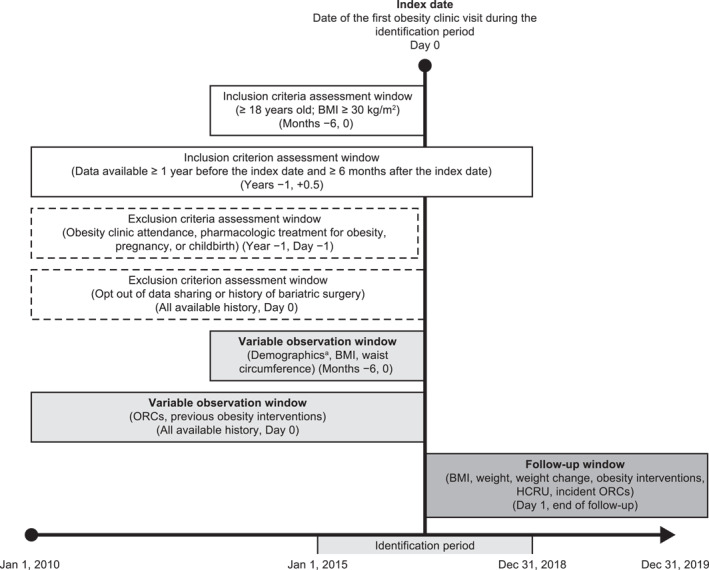
Study design. Individuals required at least 6 months of data in the data source following the index date and a minimum of 1 year of data before the index date (i.e., 1 year before and 6 months after the date of the first obesity clinic visit with BMI ≥ 30 kg/m^2^). ^a^All available records for demographic characteristics that are unlikely to change, such as ethnicity and sex, were used. BMI, body mass index; HCRU, healthcare resource utilization; ORC, obesity‐related complication.

This was an observational study using secondary data, with a study period starting on January 1, 2010 and ending on December 31, 2019 (Figure 1). Individuals aged 18 years or older with a BMI ≥ 30 kg/m^2^ who attended an obesity treatment clinic were included during an identification period starting on January 1, 2015 for SIR and January 1, 2016 for Discover, and ending on December 31, 2018. The index date was the date of the first obesity clinic visit with an eligible BMI in the Salford Royal Foundation Trust Weight Management Service (January 1, 2015–December 31, 2018) or in obesity services in North West London (January 1, 2016–December 31, 2018). BMI records in the 6 months before the index date were considered valid.

Individuals were required to have data for a minimum of 1 year before and 6 months after the index date in the databases. Individuals were excluded if they had attended an obesity clinic or received pharmacological management for obesity in the 12 months before the index date. Additional exclusion criteria were a history of metabolic/bariatric surgery at any time before the index date, documented pregnancy or childbirth in the year before the index date, and opting out of healthcare data sharing for research purposes.

### 2.3 Study Measures

Outcomes of interest were the prevalence and incidence of individual ORCs, treatment pathways, transitions between BMI groups, and costs and HCRU associated with ORCs. Baseline demographic and clinical characteristics, and obesity interventions, were assessed at the index date. The prevalence of ORCs, identified using all available linked data in the databases, was evaluated for the pre‐index period, which included the index date, and the incidence of new ORCs was calculated during the follow‐up period. The ORCs of interest were prediabetes, type 2 diabetes (T2D), dyslipidemia, hypertension, cardiovascular disease, knee osteoarthritis, musculoskeletal pain, asthma, obstructive sleep apnea, metabolic‐associated steatohepatitis/metabolic‐associated fatty liver disease, chronic kidney disease, gastroesophageal reflux disease, psoriasis, polycystic ovary syndrome, and urinary incontinence.

Lifestyle modification interventions for obesity management were recorded in the absence of a similar record in the preceding 6 months. Lifestyle modification interventions included all nonpharmacological and non‐surgical management: firstly, lifestyle programs and advice relating to weight management, diet, exercise and/or alcohol; secondly, dietetic interventions, including records of complying with dietary regimens, diabetic dietary reviews or diets related to diabetes, and dietetic appointments; and third, physical activity. Lifestyle programs included starting a weight management plan, health lifestyle programs, and attending a slimming club. Lifestyle advice included advice on diet, exercise, alcohol, and health education about lifestyle. Dietetic interventions included records of complying with dietary regimens, diabetic dietary reviews, dietetic appointments, and diets related to diabetes.

Pharmacological interventions were captured separately for the pre‐index period to summarize medical history and for the follow‐up period, using Read, Anatomical Therapeutic Chemical and British National Formulary codes. Orlistat was the only medication for obesity available in England during the study period. Metabolic and bariatric surgical interventions, namely gastric bypass, sleeve gastrectomy, and gastric banding, were captured using the OPCS Classification of Interventions and Procedures version 4 codes.

HCRU relating to obesity care and ORCs was captured. The HCRU variables captured included family physician and specialist visits, hospitalizations, emergency department visits, and length of stay. For HCRU arising from ORCs, the follow‐up period started with the time of first diagnosis of each respective ORC (i.e., the ORC index date), which could be either in the pre‐index period or during follow‐up. Healthcare costs were not directly available in the databases. Therefore, unit costs (tariffs) were sourced from public databases, including the National Health Service (NHS) Healthcare Resource Group tariffs (2022–2023) [18] and the British National Formulary for medications [19], and assigned to the HCRU from the database at an aggregate level to derive an average estimated cost per person. Healthcare costs were calculated for obesity medications and other medications of interest, concomitant medications in obesity care, laboratory tests, comorbidities of interest, and medical events during outpatient and inpatient visits.

### 2.4 Statistical Methods

For categorical variables, data are presented as numbers and percentages. For continuous variables, data are presented as mean with standard deviation (SD) or median with interquartile range (IQR). Only individuals with available data for a particular variable were included in the calculation of percentages and summary statistics.

## 3 Results

### 3.1 Baseline Characteristics and ORCs

In total, 1698 people living with obesity and attending an obesity clinic between 2015 and 2019 were included from Discover (North West London) and 561 were included from SIR (Greater Manchester). The median age was 52 years (IQR 43–61) in Discover and 55 years (IQR 42–67) in SIR (Table 1). Overall, 63.6% (*n* = 1080) and 61.3% (*n* = 344) of individuals were women, in each database, respectively. In Discover, 52.0% of individuals were White, 16.7% were Asian or Asian British, and 15.1% were Black or Black British. In SIR, these percentages were 83.6%, 1.2%, and 2.1%, respectively.

**TABLE 1:** Baseline characteristics for the study cohorts, including ORCs.

Characteristic	Discover (North West London)	SIR (Greater Manchester)
	*N* = 1698	*N* = 561
Sex, *n* (%)		
Women	1080 (63.6)	344 (61.3)
Ethnicity, *n* (%)		
White	883 (52.0)	469 (83.6)
Asian or Asian British	283 (16.7)	7 (1.2)
Black or Black British	257 (15.1)	12 (2.1)
Mixed	66 (3.9)	0 (0)
Other ethnic groups	208 (12.2)	49 (8.7)
Unknown	< 6	24 (4.3)
Age, years		
Median (IQR)	52 (43–61)	55 (42–67)
Age range, years, *n* (%)		
18–24	49 (2.9)	20 (3.6)
25–34	130 (7.7)	63 (11.2)
35–44	302 (17.8)	72 (12.8)
45–54	502 (29.6)	117 (20.9)
55–64	390 (23.0)	128 (22.8)
65 and above	325 (19.1)	161 (28.7)
BMI, kg/m^2^		
Median (IQR)	39.3 (34.2–44.9)	35.7 (31.8–42.6)
BMI classes, *n* (%)		
30 to < 35 kg/m^2^	461 (27.1)	248 (44.2)
35 to < 40 kg/m^2^	411 (24.2)	122 (21.7)
≥ 40 kg/m^2^	826 (48.6)	191 (34.0)
ORCs, pre‐index period, *n* (%)		
Hypertension	648 (38.2)	191 (34.0)
CKD	81 (4.8)	71 (12.7)
Asthma	358 (21.1)	117 (20.9)
T2D	578 (34.0)	124 (22.1)
Urinary incontinence	94 (5.5)	45 (8.0)
GERD	138 (8.1)	98 (17.5)
Musculoskeletal pain	269 (15.8)	149 (26.6)
MASH/MAFLD	135 (8.0)	47 (8.4)
Dyslipidemia	192 (11.3)	50 (8.9)
Obstructive sleep apnea	47 (2.8)	27 (4.8)
Knee osteoarthritis	72 (4.2)	35 (6.2)
Psoriasis	56 (3.3)	35 (6.2)
CHF	56 (3.3)	36 (6.4)
PCOS	38 (2.2)	13 (2.3)
Prediabetes	17 (1.0)	9 (1.6)
Acute CVD, any of	54 (3.2)	61 (10.9)
MI	12 (0.7)	33 (5.9)
Unstable angina	20 (1.2)	< 6
TIA	12 (0.7)	8 (1.4)
Stroke	14 (0.8)	24 (4.3)

Abbreviations: BMI, body mass index; CHF, chronic heart failure; CKD, chronic kidney disease; CVD, cardiovascular disease; GERD, gastroesophageal reflux disease; IQR, interquartile range; MASH/MAFLD, metabolic dysfunction‐associated steatohepatitis/metabolic dysfunction‐associated fatty liver disease; MI, myocardial infarction; ORC, obesity‐related complication; PCOS, polycystic ovary syndrome; SIR, Salford Integrated Record; T2D, type 2 diabetes; TIA, transient ischemic attack.

Median BMI was 39.3 kg/m^2^ (IQR 34.2–44.9) in Discover and 35.7 kg/m^2^ (IQR 31.8–42.6) in SIR, and 48.6% (*n* = 826) and 34.0% (*n* = 191) of individuals, respectively, had a BMI ≥ 40 kg/m^2^. Waist circumference data were available for 25.7% and 16.0% of individuals in Discover and SIR, respectively: median waist circumference was 115 cm (IQR 106–126) in Discover and 112 cm (IQR 100–123) in SIR. The most frequently recorded ORCs were hypertension (Discover: 38.2%; SIR: 34.0%), T2D (Discover: 34.0%; SIR: 22.1%), asthma (Discover: 21.1%; SIR: 20.9%), and musculoskeletal pain (Discover: 15.8%; SIR: 26.6%). Prediabetes was recorded for only 1.0% and 1.6% of individuals in Discover and SIR, respectively, likely due to under‐reporting of prediabetes in EHR data.

### 3.2 Treatment Pathways Over Follow‐Up

Lifestyle advice was the most reported intervention (Discover: 46.7% [*n* = 793]; SIR: 45.6% [*n* = 256]; Table 2). The median time to first lifestyle advice intervention was 310 days (IQR 154–568) in Discover and 359 days (IQR 139–701) in SIR. Other lifestyle modification interventions received were physical activity (Discover: 1.4% [*n* = 24]; no individuals in SIR), dietetic intervention (Discover: 3.4% [*n* = 57]; SIR: 7.0% [*n* = 39]), and lifestyle programs (Discover: 0.4% [*n* = 7]; SIR: 2.7% [*n* = 15]).

**TABLE 2:** Numbers of individuals receiving interventions, and time to interventions.

Characteristic	Discover (North West London)	SIR (Greater Manchester)
	*N* = 1698	*N* = 561
Lifestyle modification intervention		
Lifestyle advice^a^, number of individuals with prescribed interventions (%)	793 (46.7)	256 (45.6)
Time to first lifestyle advice intervention, days		
Mean (SD)	390 (302)	475 (406)
Median (IQR)	310 (154–568)	359 (139–701)
Physical activity, number of individuals with prescribed interventions (%)	24 (1.4)	0 (0)
Time to first physical activity intervention, days		
Mean (SD)	606 (349)	NA
Median (IQR)	487 (314–860)	NA
Dietetic intervention^b^, number of individuals with prescribed interventions (%)	57 (3.4)	39 (7.0)
Time to first dietetic intervention, days		
Mean (SD)	402 (293)	530 (497)
Median (IQR)	350 (174–582)	386 (90–927)
Lifestyle program^c^, number of individuals with prescribed interventions (%)	7 (0.4)	15 (2.7)
Time to first lifestyle program intervention, days		
Mean (SD)	182 (180)	423 (378)
Median (IQR)	84 (56–287)	357 (165–621)
Pharmacological weight loss intervention		
Orlistat, number of individuals (%)	124 (7.3)	67 (11.9)
Time to orlistat prescription, days		
Mean (SD)	285 (282)	303 (381)
Median (IQR)	202 (62–395)	160 (33–314)
Metabolic and bariatric procedures		
Gastric bypass, number of individuals (%)	143 (8.4)	20 (3.6)
Time to first gastric bypass, days		
Mean (SD)	642 (219)	418 (378)
Median (IQR)	671 (484–788)	276 (190–542)
Gastric banding, number of individuals (%)	19 (1.1)	0
Time to first gastric banding, days		
Mean (SD)	496 (256)	NA
Median (IQR)	461 (334–631)	NA
Sleeve gastrectomy, number of individuals (%)	101 (5.9)	8 (1.4)
Time to first sleeve gastrectomy, days		
Mean (SD)	680 (211)	631 (225)
Median (IQR)	717 (535–827)	681 (565–712)

Abbreviations: IQR, interquartile range; NA, not applicable; SD, standard deviation; SIR, Salford Integrated Record.

^a^ Lifestyle advice included advice on diet, exercise, alcohol, and health education about lifestyle.

^b^ Dietetic interventions included records of complying with dietary regimens, diabetic dietary reviews, dietetic appointments, and diets related to diabetes.

^c^ Lifestyle programs included starting a weight management plan, health lifestyle programs, and attending a slimming club.

Orlistat prescriptions were reported for 7.3% (*n* = 124) and 11.9% (*n* = 67) of individuals in Discover and SIR, respectively. The median time to first orlistat prescription was 202 days (IQR 62–395) in Discover and 160 days (IQR 33–314) in SIR. Gastric bypass was recorded for 8.4% (*n* = 143) and 3.6% (*n* = 20) of individuals in Discover and SIR, respectively, whereas sleeve gastrectomy was recorded for 5.9% (*n* = 101) and 1.4% (*n* = 8) of individuals, respectively. In total, 1.1% of individuals in Discover (*n* = 19) had gastric banding, but none were reported in SIR. The median time to first gastric bypass was 671 days (IQR 484–788) in Discover and 276 days (IQR 190–542) in SIR; median time to first sleeve gastrectomy was 717 days (IQR 535–827) and 681 days (IQR 565–712) in Discover and SIR, respectively. The numbers of individuals receiving interventions and time to interventions by BMI group are shown in Supporting Information S1: Tables S1 and S2. In both Discover and SIR, the proportion of individuals receiving metabolic/bariatric procedures was highest in the groups with BMI ≥ 40 kg/m^2^. In addition, greater proportions of individuals with BMI ≥ 35 kg/m^2^ received orlistat than those with a BMI between 30 and 35 kg/m^2^ in both databases.

Sequences of obesity management are shown in Supporting Information S1: Table S3. Most individuals received at least one intervention (Discover: *n* = 1012/1698; SIR: *n* = 323/561). Most individuals (74.9%–78.6%) received a lifestyle intervention as their first intervention. A metabolic/bariatric procedure was recorded as the first intervention for 16.3% of individuals (*n* = 165) in Discover and 6.2% (*n* = 20) of individuals in SIR. In Discover, 8.8% (*n* = 89) received a pharmacological intervention as their first intervention, compared with 15.2% (*n* = 49) in SIR. For each subsequent intervention, the proportions of individuals receiving metabolic/bariatric procedures remained relatively stable, the proportions receiving lifestyle interventions decreased, and the proportions receiving pharmacological interventions increased.

### 3.3 BMI Changes Over Follow‐Up

**FIGURE 2:**
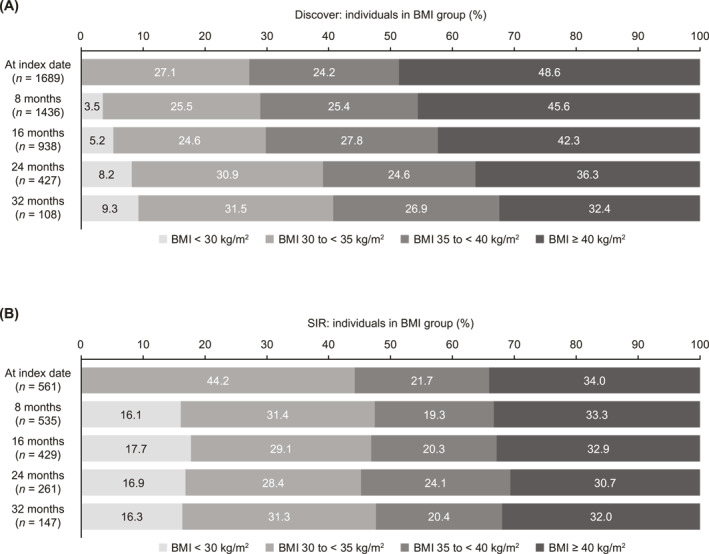
Individuals in each BMI group at index date and at subsequent time points in (A) Discover and (B) SIR databases. BMI, body mass index; SIR, Salford Integrated Record.

BMI distributions at baseline and subsequent changes differed between the databases. In Discover, nearly half of the individuals (48.6%) had BMI ≥ 40 kg/m^2^ at baseline, 24.2% had BMI 35–< 40 kg/m^2^, and 27.1% had BMI 30–< 35 kg/m^2^. In SIR, nearly half of the individuals (44.2%) had BMI 30–< 35 kg/m^2^ at baseline, 21.7% had BMI 35–< 40 kg/m^2^, and 34.0% had BMI ≥ 40 kg/m^2^ (Figure 2).

Most individuals remained in their baseline BMI groups after 8 months of follow‐up in both databases (Figure 2). However, approximately 13% of individuals in Discover and 11% in SIR who were in the BMI ≥ 40 kg/m^2^ group at baseline transitioned to a lower group during this time. Data were available for subsequent time points over follow‐up, albeit with decreasing sample sizes, allowing for mapping of trends up to 32 months. The proportion of individuals in the highest BMI group in Discover decreased steadily over follow‐up, and the proportions in the other groups increased. After 32 months, 9.3% of individuals had BMI < 30 kg/m^2^. The proportion of individuals with BMI 30–< 35 kg/m^2^ in SIR decreased markedly at 8 months to 31.4%, with 16.1% of the cohort having BMI < 30 kg/m^2^. Thereafter, the proportions of individuals in each BMI group remained relatively stable. Accordingly, the median BMI in the 35–< 40 and ≥ 40 kg/m^2^ groups decreased over time in Discover and remained similar in SIR (Supporting Information S1: Table S4).

Data on median BMI by group stratified by gastric bypass surgery (yes or no) were available in Discover, albeit with decreasing sample sizes over time. Overall, there was a notable trend for a larger decrease in BMI over time in individuals who underwent gastric bypass surgery compared with individuals who did not (Supporting Information S1: Table S4). The median (IQR) BMI decreased from 44.8 kg/m^2^ (40.0–49.1) at baseline to 34.8 kg/m^2^ (27.9–37.8) at 32 months after the index date among patients who underwent gastric bypass surgery, whereas among patients who did not undergo the surgery, the median BMI changed from 38.8 kg/m^2^ (33.9–44.2) to 36.5 kg/m^2^ (33.4–42.0).

### 3.4 HCRU Over Follow‐Up

Throughout follow‐up, primary care was the most frequent healthcare setting, with a median of 28 encounters per person (IQR 17.0–47.0) in Discover and 78 encounters per person (IQR 45.0–130.0) in SIR, whereas median numbers of encounters with other specialists were considerably lower (Supporting Information S1: Table S5). In total, 67.0% of individuals (*n* = 1137) in Discover and 65.1% in SIR (*n* = 365) experienced a hospitalization during the study period. Median length of stay was 4.0 days (IQR 2.0–8.0) in Discover and 6.0 days (IQR 2.0–19.0) in SIR (Supporting Information S1: Table S5).

Numbers of prescriptions are shown in Supporting Information S1: Table S6. Overall, 65.7% of individuals in Discover (*n* = 1115) and 72.4% in SIR (*n* = 406) received any prescription. Statins and biguanides were the most frequently prescribed medications in both Discover (*n* = 351 [20.7%] and *n* = 499 [29.4%], respectively) and SIR (*n* = 241 [43.0%] and *n* = 147 [26.2%], respectively). In total, there were 530 prescriptions for orlistat in Discover and 585 in SIR.

### 3.5 Costs and HCRU Associated With ORCs Over Follow‐Up

The overall occurrence of ORCs at baseline and over follow‐up are summarized in Supporting Information S1: Table S7 for Discover and Supporting Information S1: Table S8 for SIR. The most common incident ORCs over follow‐up were musculoskeletal pain (Discover: 6.2%; SIR: 8.9%), hypertension (Discover: 6.4%; SIR: 7.1%), and T2D (Discover: 6.4%; SIR: 6.2%).

There was wide variation in the costs related to specific ORCs, as well as disparities between the two data sources. However, the greatest direct costs were largely attributable to cardiovascular diseases in both Discover and SIR. The highest annualized costs per person related to ORCs were attributed to incident myocardial infarction (Discover: 2285 GBP; SIR: 2194 GBP), incident stroke (Discover: 3005 GBP; SIR: 1550 GBP), chronic heart failure (Discover: 2390 GBP; SIR: 2085 GBP), and diabetic foot (Discover: 4392 GBP; SIR: 1935 GBP; Table 3).

**TABLE 3:** Estimates of costs attributed to HCRU associated with ORCs and medical events.

Complication/medical event	Discover (North West London)		SIR (Greater Manchester)	
	*N* = 1698		*N* = 561	
	Number of individuals (%)	Annualized cost per person	Number of individuals (%)	Annualized cost per person
Asthma	407 (24.0)	£121	128 (22.8)	£40
CHF	**87 (5.1)**	**£2390**	**54 (9.6)**	**£2085**
CKD	125 (7.4)	£747	100 (17.8)	£299
Dyslipidemia	242 (14.3)	£288	60 (10.7)	£329
GERD	194 (11.4)	£415	131 (23.4)	£294
Hypertension	757 (44.6)	£360	231 (41.2)	£456
Knee osteoarthritis	108 (6.4)	£896	48 (8.6)	£908
Musculoskeletal pain	375 (22.1)	£156	199 (35.5)	£160
MASH/MAFLD	209 (12.3)	£123	70 (12.5)	No HCRU reported in the sample
Obstructive sleep apnea	133 (7.8)	£691	41 (7.3)	£110
PCOS	52 (3.1)	£277	14 (2.5)	£58
Prediabetes	22 (1.3)	£175	10 (1.8)	£682
Psoriasis	71 (4.2)	£230	41 (7.3)	£406
T2D	687 (40.5)	£431	159 (28.3)	£493
Urinary incontinence	136 (8.0)	£62	58 (10.3)	£84
Incident CVDs^a^				
MI (only incident)	**12 (0.7)**	**£2285**	**8 (1.4)**	**£2194**
In the 30 days after the complication		£2532		£1683
31 days to 1 year after the complication		£1221		£1308
Second year after the complication		£225		£594
Stroke (only incident)	**18 (1.1)**	**£3005**	**8 (1.4)**	**£1550**
In the 30 days after the complication		£3416		£3068
31 days to 1 year after the complication		£1350		No HCRU reported in the sample
Second year after the complication		£48		No HCRU reported in the sample
TIA (only incident)	6 (0.4)	£397	< 6	Too few to estimate
Unstable angina (only incident)	9 (0.5)	£1067	< 6	Too few to estimate
In the 30 days after the complication		£882		Too few to estimate
31 days to 1 year after the complication		£382		Too few to estimate
T2D complications				
Diabetic foot	**25 (1.5)**	**£4392**	**15 (2.7)**	**£1935**
Diabetic neuropathy	38 (2.2)	£904	< 6	Too few to estimate
Diabetic retinopathy	222 (13.1)	£976	35 (6.2)	£449

*Note:* Bold text indicates ORCs associated with the highest annualized costs per person.

Abbreviations: CHF, chronic heart failure; CKD, chronic kidney disease; CVD, cardiovascular disease; GERD, gastroesophageal reflux disease; HCRU, healthcare resource utilization; MASH/MAFLD, metabolic dysfunction‐associated steatohepatitis/metabolic dysfunction‐associated fatty liver disease; MI, myocardial infarction; ORC, obesity‐related complication; PCOS, polycystic ovary syndrome; SIR, Salford Integrated Record; T2D, type 2 diabetes; TIA, transient ischemic attack.

^a^ To estimate the cost of acute and subsequent phases, costs for incident CVDs were calculated both overall and separately by time after the complication.

## 4 Discussion

This study provided a detailed description of obesity care pathways and related costs between 2015 and 2019, characterizing people living with obesity who received care at two specialist weight management clinics in England (North West London and Greater Manchester) and addressing an evidence gap regarding obesity care pathways in the UK. As expected, many individuals entering specialist obesity care had a BMI of 35 kg/m^2^ or more (obesity class II or III), and many had ORCs. Newly diagnosed ORCs pre‐index or during follow‐up, particularly incident acute cardiovascular events such as stroke and myocardial infarction, resulted in high direct healthcare costs. Most individuals remained in their baseline BMI group during follow‐up, and just over 10% transitioned to lower groups after 8 months. Relatively few individuals received pharmacological or surgical intervention in the study cohorts, and the average time to first intervention was considerable, taking 6 months or more.

The results of this study regarding lifestyle advice and pharmacological intervention aligned with published retrospective evidence from UK primary care. A study of individuals with obesity in the period 2010–2019 reported similar rates of lifestyle interventions and medication to those observed in the present study [8]. In a separate UK cohort with overweight or obesity (2005–2012), advice was the most recorded intervention in the lower BMI classes, whereas medication was the most common intervention in women with BMI ≥ 35 kg/m^2^ and in men with BMI ≥ 40 kg/m^2^ [9], which aligns with the present study. Rates of prescription medications for weight management were also similar to those reported in the European RESOURCE survey [13]. As the present study focused specifically on people receiving specialist obesity care, rates of metabolic and bariatric surgery were higher than previously reported in English primary care [11, 12] and in the RESOURCE survey [13], in which BMI was self‐reported.

The present study highlights a trend of relatively long waiting times for metabolic and bariatric surgery in the UK, relative to other countries. A survey of expert surgical representatives across Europe in 2016–2017 indicated that waiting times varied widely between countries, ranging from a period of months to more than a year [20]. In the USA, the average waiting time for metabolic/bariatric surgery was reported as 209 days (approximately 7 months) in a study conducted during 2014–2015 [21], and 159 days (approximately 5.5 months) by the end of a study that finished in 2016 [22]. In contrast, a previous study of an English cohort who received metabolic/bariatric surgery found that only 36% underwent surgery less than 2 years after becoming eligible, and 37% did not undergo surgery until more than 4 years had elapsed since the time that they first became eligible [12]. In the present study, time to surgery varied between approximately 9 months and 2 years, depending on the procedure type and study site. Disparities between the UK and other countries can be explained in part by limitations in the provision of both specialist obesity management services and metabolic and bariatric surgery services [6, 10].

This study was designed to assess trends in obesity specialist care in the period before tirzepatide and semaglutide 2.4 mg became available in the UK. Plans for phased roll‐outs for new medications have been developed, meaning that medications initially available only for individuals with BMI ≥ 40 kg/m^2^ and three ORCs will become available to wider populations [5, 14, 23, 24]. As these treatments become more widely available, it is anticipated that the obesity care landscape in the UK will shift considerably. Once sufficient time has elapsed, updated complementary analyses will be valuable to assess the impact of newer medications on treatment pathways and outcomes.

A key strength of this study was the use of the Discover and SIR databases. Discover accounts for more than 95% of the North West London population and SIR includes nearly all patients in the Salford region in Greater Manchester, North West England, starting from 2008 [15, 17]. In addition to this excellent population coverage, both databases link primary and secondary care data, meaning that multiple outcomes throughout the obesity care pathway could be assessed, building a holistic picture of treatment journeys. By using data from two treatment centers, the study took into account regional disparities within England. However, it should be noted that the results of this study were not representative of all UK nations and regions, particularly given that provision of weight management services varies across the UK [5, 6].

Only NHS data were included in this study, and treatment pathways in private care may differ. Furthermore, the availability of data for each individual varied, and continuity of records might have been incomplete for some individuals if they sought healthcare in another facility. As this was an observational study, data collection reflected routine clinical practice rather than mandatory assessments at pre‐specified time points, which affected the availability of data and their interpretation, and could have increased the potential for measurement error. Although trends in BMI changes over the follow‐up period were consistent for both databases, they should be treated with caution because missing values increased over time. In addition, waist circumference data were available for only a minority of individuals, meaning that the study relied on BMI as a measure of obesity.

For some endpoints with limited availability, it is difficult to distinguish between limitations in recording and lack of, or inertia in, treatment. For example, data on treatment pathways recording metabolic/bariatric surgery as the first intervention in 16.3% of individuals in Discover and 6.2% in SIR indicated that some individuals were likely to have received previous treatment for obesity before inclusion in this study, which is not captured in the available records; alternatively, such individuals may have received specialist care late in their disease course. Although the study was descriptive, it may also have been prone to selection bias and confounding. Prediabetes was recorded for only 1%–2% of the included individuals, likely due to under‐reporting of prediabetes in EHR data. The UK Office for National Statistics has estimated the prevalence of prediabetes as 10.2% in people living with overweight and 14.8% in people living with obesity in the UK [25]. Other sources of selection bias and confounding may arise via under‐reporting of lifestyle interventions in EHRs, and owing to the transition from paper to electronic health records. Additionally, the costing analysis for ORCs was limited by small sample sizes, particularly for incident cardiovascular disease.

In conclusion, both cohorts had a high comorbidity burden, including hypertension, T2D, musculoskeletal pain, and chronic pulmonary diseases. Few individuals received pharmacological treatments and metabolic/bariatric surgical procedures, and median times to first intervention of any type were considerable, typically more than 6 months. A substantial proportion of individuals remained in their original BMI class over time, even up to 32 months of follow‐up, including those in the highest BMI group. The direct costs associated with treating ORCs in obesity were substantial. Taken together, these data indicate a high unmet need for individuals living with obesity in England.

## Author Contributions

C.S.M. and V.A. carried out the study design. S.M. and B.W. acquired and analyzed the data. All authors contributed to data interpretation, drafting, and critical revision of the manuscript text, and approved the manuscript for submission.

## Funding

This study, medical writing and editorial support were funded by Novo Nordisk A/S.

## Conflicts of Interest

A.D.M. has received research funding from Anabio, Boehringer Ingelheim, the European Union, Fractyl, Gila, Jon Moulton Charitable Foundation, the Medical Research Council, the National Institute for Health and Care Research, Novo Nordisk, and Randox. He has received honoraria for lectures and presentations from Algorithm, AstraZeneca, Boehringer Ingelheim, Currax Pharmaceuticals, Eli Lilly, Ethicon, GI Dynamics, Medtronic, Novo Nordisk, and Screen Health. A.D.M. is a shareholder in the Beyond BMI clinic, which provides clinical obesity care. S.C. and C.S.M. are employees and shareholders of Novo Nordisk A/S. L.M.H. is an employee and shareholder of Novo Nordisk Denmark. V.A. is an employee of Novo Nordisk UK. S.M. is an employee of NorthWest EHealth Ltd and B.W. was an employee of NorthWest EHealth Ltd at the time of study conduct; NorthWest EHealth Ltd was responsible for the acquisition, analysis, and interpretation of data in the study. L.Z. and J.C.S. are employees of IQVIA, which was the contract research organization responsible for implementing the study.

## Supporting information


Supporting Information S1

## Data Availability

The authors confirm that all data supporting the findings of this study are available in the article and its supplementary materials. Discover does not permit the sharing or publication of individual patient data. Information on how to access Discover for research purposes can be found here: https://discover‐now.co.uk/how‐to‐access‐the‐data/. SIR does not permit the sharing or publication of individual patient data. The SIR database is now integrated with the Greater Manchester (GM) Secure Data Environment (SDE) and information on how to access the data for research purposes can be found here: https://healthinnovationmanchester.com/gms‐secure‐data‐environment‐sde‐for‐health‐and‐care/.
