# Supplementary material for: Mapping Obesity Care Pathways and Healthcare Resource Use From 2015 to 2019 in England
Source: Obes Sci Pract. 2026 Jul 19;12(4):e70173. doi: 10.1002/osp4.70173 (PMC13382353; doi:10.1002/osp4.70173)
Supplement: Supplementary file 1 — Supporting Information S1 [file OSP4-12-e70173-s001.docx]

**Online Supporting Information**

**Mapping Obesity Care Pathways and Healthcare Resource Use From 2015 to 2019 in England**

Alexander Dimitri Miras^1,2^ | Silvia Capucci^3^ | Sylwia Migas^4^ | Bozydar Wrona^4^ | Liwei Zhao^5^ | Johann Castañeda-Sanabria^6^ | Lise M. Hagelund^7^ | Vasileios Antavalis^8^ | Camilla S. Morgen^3^

^1^Imperial College London, Hammersmith Hospital, London, UK

^2^School of Medicine, Ulster University, Derry, UK

^3^Novo Nordisk A/S, Søborg, Denmark

^4^NorthWest EHealth Ltd, Manchester, UK

^5^IQVIA, Mölndal, Sweden

^6^IQVIA, London, UK

^7^Novo Nordisk, Copenhagen, Denmark

^8^Novo Nordisk, Gatwick, UK

**Correspondence**

Alexander Dimitri Miras, School of Medicine, Ulster University, Northland Road, Derry, BT48 7JL, UK.

Email: [A.Miras@ulster.ac.uk](mailto:A.Miras@ulster.ac.uk)

Telephone: 07958 377674

# Supporting Information

## Table S1 Numbers of individuals receiving interventions, and time to interventions by BMI class at baseline in Discover (North West London).

|  | **BMI group at baseline** | | | |
| --- | --- | --- | --- | --- |
| **Characteristic** | **Overall**  ***N* = 1698** | **30 to < 35 kg/m^2^,**  ***n* = 461** | **35 to < 40 kg/m^2^,**  ***n* = 411** | **≥ 40 kg/m^2^,**  ***n* = 826** |
| **Lifestyle modification intervention** |  |  |  |  |
| **Lifestyle advice, number of individuals with prescribed interventions (%)** | 793 (46.7) | 210 (45.5) | 205 (49.9) | 378 (45.8) |
| **Time to first lifestyle advice intervention, days** | | | | |
| Mean (SD) | 390 (302) | 382 (286) | 361 (293) | 410 (314) |
| Median (IQR) | 310  (154–568) | 300  (161–561) | 288  (126–545) | 326  (155–593) |
| **Physical activity, number of individuals with prescribed interventions (%)** | 24 (1.4) | < 6 | DCS | 11 (1.3) |
| **Time to first physical activity intervention, days** | | | | |
| Mean (SD) | 606 (349) | 395 (341) | 748 (381) | 567 (301) |
| Median (IQR) | 487  (314–860) | 275  (182–488) | 752  (461–936) | 479  (328–838) |
| **Dietetic intervention, number of individuals with prescribed interventions (%)** | 57 (3.4) | 9 (2.0) | 17 (4.1) | 31 (3.8) |
| **Time to first dietetic intervention, days** | | | | |
| Mean (SD) | 402 (293) | 348 (246) | 439 (320) | 397 (296) |
| Median (IQR) | 350  (174–582) | 325  (162–464) | 406  (229–636) | 350  (172–575) |
| **Lifestyle programs, number of individuals with prescribed interventions (%)** | 7 (0.4) | 0 | < 6 | < 6 |
| **Time to first lifestyle program intervention, days** | | | | |
| Mean (SD) | 182 (180) | NA | 186 (184) | 179 (205) |
| Median (IQR) | 84 (56–287) | NA | 84 (80–241) | 106 (36–249) |
| **Pharmacological weight loss intervention** |  |  |  |  |
| **Orlistat, number of individuals (%)** | 124 (7.3) | 16 (3.5) | 31 (7.5) | 77 (9.3) |
| **Time to orlistat initiation, days** | | | | |
| Mean (SD) | 285 (282) | 277 (248) | 330 (318) | 268 (275) |
| Median (IQR) | 202  (62–395) | 201  (106–360) | 231  (67–597) | 200  (59–350) |
| **Bariatric procedures** |  |  |  |  |
| **Gastric bypass, number of individuals (%)** | 143 (8.4) | 8 (1.7) | 26 (6.3) | 109 (13.2) |
| **Time to first gastric bypass, days** | | | | |
| Mean (SD) | 642 (219) | 641 (277) | 654 (197) | 639 (222) |
| Median (IQR) | 671  (484–788) | 608  (404–882) | 667  (599–762) | 671  (477–790) |
| **Gastric banding, number of individuals (%)** | 19 (1.1) | 0 | < 6 | DCS |
| **Time to first gastric banding, days** | | | | |
| Mean (SD) | 496 (256) | NA | 504 (101) | 494 (279) |
| Median (IQR) | 461  (334–631) | NA | 517  (458–558) | 460  (306–691) |
| **Sleeve gastrectomy, number of individuals (%)** | 101 (5.9) | < 6 | DCS | 78 (9.4) |
| **Time to first sleeve gastrectomy, days** | | | | |
| Mean (SD) | 680 (211) | 752 (241) | 644 (253) | 688 (199) |
| Median (IQR) | 717  (535–827) | 752  (667–838) | 568  (470–792) | 731  (570–827) |

Abbreviations: BMI, body mass index; DCS, double count suppression; IQR, interquartile range; NA, not applicable; SD, standard deviation.

## Table **S2** Numbers of individuals receiving interventions, and time to interventions by BMI class at baseline in SIR (Greater Manchester).

|  | **BMI group at baseline** | | | |
| --- | --- | --- | --- | --- |
| **Characteristic** | **Overall**  ***N* = 561** | **30 to < 35 kg/m^2^,**  ***n* = 248** | **35 to < 40 kg/m^2^,**  ***n* = 122** | **≥ 40 kg/m^2^,**  ***n* = 191** |
| **Lifestyle modification intervention** |  |  |  |  |
| **Lifestyle advice, number of individuals with prescribed interventions (%)** | 256 (45.6) | 104 (41.9) | 53 (43.4) | 99 (51.8) |
| **Time to first lifestyle advice intervention, days** | | | | |
| Mean (SD) | 475 (406) | 535 (463) | 412 (328) | 446 (375) |
| Median (IQR) | 359  (139–701) | 410  (132–799) | 327  (124–630) | 332  (142–650) |
| **Physical activity, number of individuals with prescribed interventions (%)** | NR | NR | NR | NR |
| **Time to first physical activity intervention, days** | | | | |
| Mean (SD) | NR | NR | NR | NR |
| Median (IQR) | NR | NR | NR | NR |
| **Dietetic intervention, number of individuals with prescribed interventions (%)** | 39 (7.0) | 19 (7.7) | < 6 | DCS |
| **Time to first dietetic intervention, days** | | | | |
| Mean (SD) | 530 (497) | 617 (556) | 414 (228) | 458 (487) |
| Median (IQR) | 386  (90–927) | 558  (109–1068) | 410  (386–593) | 295  (68–617) |
| **Lifestyle programs, number of individuals with prescribed interventions (%)** | 15 (2.7) | Statistics masked^a^ | < 6 | Statistics masked^a^ |
| **Time to first lifestyle program intervention, days** | | | | |
| Mean (SD) | 423 (378) | 505 (447) | Statistics masked^a^ | 316 (327) |
| Median (IQR) | 357  (165–621) | 495  (165–670) | Statistics masked^a^ | 280  (96–385) |
| **Pharmacological weight loss intervention** |  |  |  |  |
| **Orlistat, number of individuals (%)** | 67 (11.9) | 17 (6.9) | 21 (17.2) | 29 (15.2) |
| **Time to orlistat initiation, days** | | | | |
| Mean (SD) | 303 (381) | 455 (468) | 221 (392) | 274 (295) |
| Median (IQR) | 160  (33–314) | 251  (87–849) | 83  (28–198) | 189  (28–336) |
| **Bariatric procedures** |  |  |  |  |
| **Gastric bypass, number of individuals (%)** | 20 (3.6) | < 6 | < 6 | 17 (8.9) |
| **Time to first gastric bypass, days** | | | | |
| Mean (SD) | 418 (378) | Statistics masked^a^ | 212 (44) | 448 (404) |
| Median (IQR) | 276  (190–542) | Statistics masked^a^ | 212  (197–228) | 282  (193– 552) |
| **Gastric banding, number of individuals (%)** | NR | NR | NR | NR |
| **Time to first gastric banding, days** | | | | |
| Mean (SD) | NR | NR | NR | NR |
| Median (IQR) | NR | NR | NR | NR |
| **Sleeve gastrectomy, number of individuals (%)** | 8 (1.4) | – | – | 8 (4.2) |
| **Time to first sleeve gastrectomy, days** | | | | |
| Mean (SD) | 631 (225) | – | – | 631 (225) |
| Median (IQR) | 681  (565–712) | – | – | 681  (565–712) |

Abbreviations: BMI, body mass index; IQR, interquartile range; NR, not reported; SD, standard deviation; SIR, Salford Integrated Record.

^a^The statistics were masked to avoid revealing other masked numbers and to protect patient privacy.

## Table S3 Obesity management sequences among individuals who received an intervention.

|  | **Discover**  **(North West London)**  ***N* = 1698** | **SIR (Greater Manchester)**  ***N* = 561** |
| --- | --- | --- |
| **First obesity intervention, n (%)** | ***n* = 1012** | ***n* = 323** |
| Bariatric procedure | 165 (16.3) | 20 (6.2) |
| Lifestyle modification | 758 (74.9) | 254 (78.6) |
| Pharmacological intervention | 89 (8.8) | 49 (15.2) |
| **Second obesity intervention, n (%)** | ***n* = 463** | ***n* < 166** |
| Bariatric procedure | 63 (13.6) | < 6 (< 4) |
| Lifestyle modification | 347 (74.9) | 124 (< 75) |
| Pharmacological intervention | 53 (11.4) | 36 (< 22) |
| **Third obesity intervention, n (%)** | ***n* = 174** | ***n* < 94** |
| Bariatric procedure | 28 (16.1) | < 6 (< 7) |
| Lifestyle modification | 111 (63.8) | 58 (< 62) |
| Pharmacological intervention | 35 (20.1) | 30 (< 32) |
| **Subsequent obesity interventions, n (%)** | ***n* = 100** | ***n* < 74** |
| Subsequent bariatric procedure | 9 (9.0) | < 6 (< 9) |
| Subsequent lifestyle modification | 60 (60.0) | 43 (< 59) |
| Subsequent pharmacological intervention | 31 (31.0) | 25 (< 34) |

Abbreviation: SIR, Salford Integrated Record.

## Table S4 Median BMI over follow-up by BMI group at baseline and by gastric bypass surgery.

|  | **BMI group** | | | | **Gastric bypass surgery** | |
| --- | --- | --- | --- | --- | --- | --- |
|  | **Overall** | **30 to < 35 kg/m^2^** | **35 to < 40 kg/m^2^** | **≥ 40 kg/m^2^** | **Yes** | **No** |
| **Discover (North West London)** | | | | |  |  |
| Baseline (*N* = 1698) | 39.3 (34.2–44.9) | 32.0 (31.1–33.3) | 37.0 (35.7–38.3) | 45.1 (42.0–49.4) | 44.8 (40.0–49.1) | 38.8 (33.9–44.2) |
| 8 months (*n* = 1436) | 38.5 (33.8–44.6) | 31.7 (30.4–33.4) | 36.9 (35.2–38.1) | 44.8 (41.2–49.7) | 44.3 (39.8–49.8) | 37.9 (33.5–43.6) |
| 16 months (*n* = 938) | 38.0 (33.7–44.1) | 31.6 (30.3–33.4) | 36.6 (34.6–38.4) | 44.0 (39.8–48.0) | 40.6 (35.0–46.4) | 37.8 (33.6–43.7) |
| 24 months (*n* = 427) | 36.2 (32.7–42.0) | 32.2 (30.6–33.8) | 35.7 (33.1–37.8) | 41.9 (37.2–47.5) | 34.8 (30.1–42.9) | 36.3 (32.9–41.9) |
| 32 months (*n* = 108) | 36.0 (32.6–41.7) | 32.9 (32.0–34.7) | 33.8 (30.4–36.8) | 40.0 (36.0–44.9) | 34.8 (27.9–37.8) | 36.5 (33.4–42.0) |
| **SIR (Greater Manchester)** | | | | |  |  |
| Baseline (*N* = 561) | 35.7 (31.8–42.6) | 31.4 (30.4–32.9) | 36.9 (35.7–38.1) | 45.8 (42.4–52.0) | NR | NR |
| 8 months (*n* = 535) | 35.2 (30.9–41.5) | 30.9 (28.5–32.7) | 36.0 (34.5–37.9) | 44.6 (40.9–50.4) | NR | NR |
| 16 months (*n* = 429) | 35.2 (31.0–41.9) | 31.0 (27.7–33.4) | 35.8 (33.3–37.6) | 44.4 (40.2–49.6) | NR | NR |
| 24 months (*n* = 261) | 35.1 (30.9–41.2) | 31.1 (28.1–33.7) | 35.1 (32.7–37.5) | 44.5 (39.3–49.3) | NR | NR |
| 32 months (*n* = 147) | 35.1 (31.5–43.6) | 31.6 (28.2–33.5) | 35.2 (32.6–37.0) | 46.3 (39.5–50.2) | NR | NR |

Data are median BMI, kg/m^2^ (IQR).

Abbreviations: BMI, body mass index; IQR, interquartile range; NR, not reported; SIR, Salford Integrated Record.

## Table S5 Numbers of healthcare visits for the study cohorts.

| **Healthcare visits** | **Discover**  **(North West London)**  ***N* = 1698** | **SIR**  **(Greater Manchester)**  ***N* = 561** |
| --- | --- | --- |
| **Person-years** | | |
| Total | 4039.5 | 1665.6 |
| Median (IQR) | 2.4 (1.7–3.1) | 3.0 (2.0–4.1) |
| **Number of individuals with any healthcare visits, n (%)** | 1698 (100.0) | 561 (100.0) |
| **Primary care encounters/contacts** | | |
| Individuals with any relevant  encounters/contacts, n (%) | 1690 (99.5) | 561 (100.0) |
| Total encounters/contacts | 61 692 | 58 891 |
| Median (IQR), per person | 28.0 (17.0–47.0) | 78.0 (45.0–130.0) |
| **Endocrinologist** | | |
| Individuals with any visits, n (%) | 783 (46.1) | 310 (55.3) |
| Total visits | 4678 | 1773 |
| Median (IQR), per person | 4.0 (2.0–8.0) | 4.0 (2.0–8.0) |
| **General surgeon** | | |
| Individuals with any visits, n (%) | 892 (52.5) | 239 (42.6) |
| Total visits | 5331 | 708 |
| Median (IQR), per person | 4.0 (2.0–8.0) | 2.0 (1.0–3.0) |
| **Cardiologist** | | |
| Individuals with any visits, n (%) | 341 (20.1) | 247 (44.0) |
| Total visits | 999 | 1061 |
| Median (IQR), per person | 2.0 (1.0–4.0) | 2.0 (1.0–4.0) |
| **Other specialist visits** | | |
| Individuals with any visits, n (%) | 1485 (87.5) | 533 (95.0) |
| Total visits | 18 691 | 22 281 |
| Median (IQR), per person | 8.0 (3.0–16.0) | 18.0 (6.0–42.0) |
| **In-person hospitalizations** | | |
| Individuals with any visits, n (%) | 1137 (67.0) | 365 (65.1) |
| Total visits | 3697 | 2356 |
| Median (IQR), per person | 2.0 (1.0–4.0) | 2.0 (1.0–5.0) |
| **Length of stay in hospital, days** | | |
| Total visits | 12 177 | 8125 |
| Median (IQR), per person | 4.0 (2.0–8.0) | 6.0 (2.0–19.0) |
| **Intensive care hospitalizations** | | |
| Individuals with any visits, n (%) | 62 (3.7) | 46 (8.2) |
| Total visits | 66 | 59 |
| Median (IQR), per person | 1.0 (1.0–1.0) | 1.0 (1.0–1.0) |
| **Length of stay in intensive care unit, days** | | |
| Total length | 436 | 369 |
| Median (IQR), per person | 4.0 (2.0–7.0) | 2.0 (1.0–5.8) |
| **Emergency department visits** | | |
| Individuals with any visits, n (%) | 1081 (63.7) | 340 (60.6) |
| Total visits | 3584 | 1277 |
| Median (IQR), per person | 2.0 (1.0–4.0) | 2.0 (1.0–4.0) |

Note: primary care encounters in SIR include routine reviews, prescriptions, text messages, letter reviews, telephone calls, and face-to-face contacts. Primary care encounters in Discover are comparatively limited, e.g., visits related to only prescribing of medication are not reported.

Abbreviations: IQR, interquartile range; SIR, Salford Integrated Record.

## Table S6 Prescriptions for medications during follow-up.

|  | **Discover**  **(North West London)**  ***N* = 1698** | **SIR (Greater Manchester)**  ***N* = 561** |
| --- | --- | --- |
| **Number of individuals with any medication prescriptions, n (%)** | 1115 (65.7) | 406 (72.4) |
| **Statins** | | |
| Individuals with any prescriptions, n (%) | 351 (20.7) | 241 (43.0) |
| Total number of prescriptions | 6691 | 11 456 |
| Mean number of prescriptions (SD)  per person | 19.1 (27.6) | 47.5 (50.9) |
| Median number of prescriptions (IQR) per person | 11.0 (6.0–19.0) | 28.0 (17.0–56.0) |
| Min–max | 1–213 | 1–242 |
| **Biguanides** | | |
| Individuals with any prescriptions, n (%) | 499 (29.4) | 147 (26.2) |
| Total number of prescriptions | 9102 | 5218 |
| Mean number of prescriptions (SD) per person | 18.2 (27.3) | 35.5 (42.5) |
| Median number of prescriptions (IQR) per person | 10.0 (5.5–19.0) | 24.0 (11.5–38.5) |
| Min–max | 1–212 | 1–234 |
| **Sulfonylureas** | | |
| Individuals with any prescriptions, n (%) | 168 (9.9) | 39 (7.0) |
| Total number of prescriptions | 3146 | 1354 |
| Mean number of prescriptions (SD) per person | 18.7 (28.1) | 34.7 (47.7) |
| Median number of prescriptions (IQR) per person | 11.0 (5.0–21.0) | 15.0 (6.5–42.5) |
| Min–max | 1–179 | 1–237 |
| **DPP-4 inhibitors** | | |
| Individuals with any prescriptions, n (%) | 186 (11.0) | 46 (8.2) |
| Total number of prescriptions | 3699 | 1685 |
| Mean number of prescriptions (SD) per person | 19.9 (31.4) | 36.6 (51.0) |
| Median number of prescriptions (IQR) per person | 9.0 (5.0–18.0) | 19.5 (6.5–29.5) |
| Min–max | 1–153 | 1–216 |
| **Thiazolidinediones** | | |
| Individuals with any prescriptions, n (%) | 19 (1.1) | 21 (3.7) |
| Total number of prescriptions | 333 | 801 |
| Mean number of prescriptions (SD) per person | 17.5 (16.2) | 38.1 (47.4) |
| Median number of prescriptions (IQR) per person | 13.0 (7.0–24.5) | 18.0 (3.0–54.0) |
| Min–max | 2–61 | 1–182 |
| **SGLT2 inhibitors** | | |
| Individuals with any prescriptions, n (%) | 134 (7.9) | 54 (9.6) |
| Total number of prescriptions | 2040 | 1435 |
| Mean number of prescriptions (SD) per person | 15.2 (19.6) | 26.6 (32.4) |
| Median number of prescriptions (IQR) per person | 9.0 (3.0–20.8) | 15.5 (5.0–33.8) |
| Min–max | 1–131 | 1–144 |
| **GLP-1RAs** | | |
| Individuals with any prescriptions, n (%) | 102 (6.0) | 16 (2.9) |
| Total number of prescriptions | 1348 | 210 |
| Mean number of prescriptions (SD) per person | 13.2 (12.6) | 13.1 (11.6) |
| Median number of prescriptions (IQR) per person | 9.0 (4.2–18.8) | 9.5 (5.5–16.8) |
| Min–max | 1–61 | 1–42 |
| **Liraglutide** | | |
| Individuals with any prescriptions, n (%) | 104 (6.1) | 20 (3.6) |
| Total number of prescriptions | 1312 | 228 |
| Mean number of prescriptions (SD) per person | 12.6 (8.8) | 11.4 (9.5) |
| Median number of prescriptions (IQR) per person | 11.0 (5.0–16.0) | 8.5 (4.0–15.8) |
| Min–max | 1–39 | 1–33 |
| **Short-acting insulin** | | |
| Individuals with any prescriptions, n (%) | 71 (4.2) | 10 (1.8) |
| Total number of prescriptions | 1015 | 113 |
| Mean number of prescriptions (SD) per person | 14.3 (10.8) | 11.3 (10.3) |
| Median number of prescriptions (IQR) per person | 10.0 (6.0–22.0) | 8.5 (3.0–17.5) |
| Min–max | 1–39 | 1–28 |
| **Intermediate-acting insulin** | | |
| Individuals with any prescriptions, n (%) | 47 (2.8) | 29 (5.2) |
| Total number of prescriptions | 588 | 517 |
| Mean number of prescriptions (SD) per person | 12.5 (13.7) | 17.8 (17.0) |
| Median number of prescriptions (IQR) per person | 6.0 (3.0–16.0) | 13.0 (6.0–22.0) |
| Min–max | 1–58 | 1–66 |
| **Long-acting insulin** | | |
| Individuals with any prescriptions, n (%) | 75 (4.4) | 12 (2.1) |
| Total number of prescriptions | 1146 | 184 |
| Mean number of prescriptions (SD) per person | 15.3 (11.1) | 15.3 (12.7) |
| Median number of prescriptions (IQR) per person | 13.0 (6.0–21.5) | 8.0 (7.0–21.5) |
| Min–max | 1–46 | 1–40 |
| **Beta blockers** | | |
| Individuals with any prescriptions, n (%) | 324 (19.1) | 180 (32.1) |
| Total number of prescriptions | 7632 | 7583 |
| Mean number of prescriptions (SD) per person | 23.6 (36.0) | 42.1 (58.2) |
| Median number of prescriptions (IQR) per person | 11.0 (5.0–22.2) | 24.0 (8.0–46.3) |
| Min–max | 1–210 | 1–452 |
| **Angiotensin-converting enzyme inhibitors** | | |
| Individuals with any prescriptions, n (%) | 465 (27.4) | 149 (26.6) |
| Total number of prescriptions | 9584 | 6676 |
| Mean number of prescriptions (SD) per person | 20.6 (30.6) | 44.8 (53.0) |
| Median number of prescriptions (IQR) per person | 12.0 (7.0–20.0) | 28.0 (12.0–57.0) |
| Min–max | 1–301 | 1–350 |
| **Other antihypertensives (angiotensin receptor agonists)** | | |
| Individuals with any prescriptions, n (%) | 288 (17.0) | 65 (11.6) |
| Total number of prescriptions | 6159 | 1838 |
| Mean number of prescriptions (SD) per person | 21.4 (30.0) | 28.3 (28.6) |
| Median number of prescriptions (IQR) per person | 13.0 (7.0–21.2) | 21.0 (12.0–33.0) |
| Min–max | 1–210 | 1–174 |
| **Alpha-adrenoceptor blockers** | | |
| Individuals with any prescriptions, n (%) | 134 (7.9) | 41 (7.3) |
| Total number of prescriptions | 2853 | 1752 |
| Mean number of prescriptions (SD) per person | 21.3 (30.7) | 42.7 (57.3) |
| Median number of prescriptions (IQR) per person | 12.0 (6.0–21.8) | 19.0 (7.0–52.0) |
| Min–max | 1–187 | 1–234 |
| **Orlistat** | | |
| Individuals with any prescriptions, n (%) | 124 (7.3) | 67 (11.9) |
| Total number of prescriptions | 530 | 585 |
| Mean number of prescriptions (SD) per person | 4.3 (5.8) | 8.7 (12.4) |
| Median number of prescriptions (IQR) per person | 2.0 (1.0–5.0) | 3.0 (1.0–11.5) |
| Min–max | 1–33 | 1–73 |
| **Cholesterol absorption inhibitors** | | |
| Individuals with any prescriptions, n (%) | 42 (2.5) | 7 (1.2) |
| Total number of prescriptions | 1126 | 367 |
| Mean number of prescriptions (SD) per person | 26.8 (43.1) | 52.4 (48.0) |
| Median number of prescriptions (IQR) per person | 9.5 (3.0–21.5) | 41.0 (18.0–68.0) |
| Min–max | 1–167 | 10–144 |

Abbreviations: DPP-4, dipeptidyl-peptidase 4; GLP-1RA, glucagon-like peptide-1 receptor agonist; IQR, interquartile range; SD, standard deviation; SGLT2, sodium-glucose cotransporter-2; SIR, Salford Integrated Record.

## Table S7 ORCs and medical events at index date and over follow-up, Discover (North West London).

|  | **At or before index** | **Follow-up, incident** | **Overall number of cases before and during follow-up** |
| --- | --- | --- | --- |
| **Characteristic** | ***N* = 1698** | ***N* = 1698** | ***N* = 1698** |
| **Complications, n (%)** | | | |
| **Individuals with any complications^a^** | 1355 (79.7) | 651 (38.3) | 1466 (86.3) |
| Asthma | 358 (21.1) | 49 (2.9) | 407 (24.0) |
| CHF | 56 (3.3) | 31 (1.8) | 87 (5.1) |
| CKD | 81 (4.8) | 44 (2.6) | 125 (7.4) |
| Dyslipidemia | 192 (11.3) | 50 (2.9) | 242 (14.3) |
| GERD | 138 (8.1) | 56 (3.3) | 194 (11.4) |
| Gout | 62 (3.7) | 17 (1.0) | 79 (4.7) |
| Hypertension | 648 (38.2) | 109 (6.4) | 757 (44.6) |
| Knee osteoarthritis | 72 (4.2) | 36 (2.1) | 108 (6.4) |
| MASH/MAFLD | 135 (8.0) | 74 (4.4) | 209 (12.3) |
| Musculoskeletal pain | 269 (15.8) | 106 (6.2) | 375 (22.1) |
| Obstructive sleep apnea | 47 (2.8) | 86 (5.1) | 133 (7.8) |
| PCOS | 38 (2.2) | 14 (0.8) | 52 (3.1) |
| Prediabetes | 17 (1.0) | < 6 | 22 (1.3) |
| Psoriasis | 56 (3.3) | 15 (0.9) | 71 (4.2) |
| T2D | 578 (34.0) | 109 (6.4) | 687 (40.5) |
| Urinary incontinence | 94 (5.5) | 42 (2.5) | 136 (8.0) |
| **CVD, any of^a^** | 54 (3.2) | 41 (2.4) | 91 (5.4) |
| MI | 12 (0.7) | 12 (0.7) | 24 (1.4) |
| Unstable angina | 20 (1.2) | 9 (0.5) | 29 (1.7) |
| TIA | 12 (0.7) | 6 (0.4) | 18 (1.1) |
| Stroke | 14 (0.8) | 18 (1.1) | 32 (1.9) |
| **T2D microvascular complications, any of^a^** | 185 (10.9) | 82 (4.8) | 257 (15.1) |
| Diabetic nephropathy | < 6 | – | < 6 |
| Diabetic retinopathy | 164 (9.7) | 58 (3.4) | 222 (13.1) |
| Diabetic neuropathy | 26 (1.5) | 12 (0.7) | 38 (2.2) |
| Diabetic foot | 12 (0.7) | 13 (0.8) | 25 (1.5) |

Abbreviations: CHF, chronic heart failure; CKD, chronic kidney disease; CVD, cardiovascular disease; GERD, gastroesophageal reflux disease; MASH/MAFLD, metabolic dysfunction-associated steatohepatitis/metabolic dysfunction-associated fatty liver disease; MI, myocardial infarction; ORC, obesity-related complication; PCOS, polycystic ovary syndrome; T2D, type 2 diabetes; TIA, transient ischemic attack.

^a^The ‘overall number of cases before and during follow-up’ may be smaller than the sum of cases ‘at or before index’ and ‘follow-up, incident’ since individuals with more than one complication/medical event would only be counted once.

## Table S8 ORCs and medical events at index date and over follow-up, SIR (Greater Manchester).

|  | **At or before index** | **Follow-up, incident** | **Overall number of cases before and during follow-up** |
| --- | --- | --- | --- |
| **Characteristic** | ***N* = 561** | ***N* = 561** | ***N* = 561** |
| **Complications, n (%)** | | | |
| **Individuals with any complications^a^** | 465 (82.9) | 243 (43.3) | 504 (89.8) |
| Asthma | 117 (20.9) | 11 (2.0) | 128 (22.8) |
| CHF | 36 (6.4) | 18 (3.2) | 54 (9.6) |
| CKD | 71 (12.7) | 29 (5.2) | 100 (17.8) |
| Dyslipidemia | 50 (8.9) | 10 (1.8) | 60 (10.7) |
| GERD | 98 (17.5) | 33 (5.9) | 131 (23.4) |
| Gout | 32 (5.7) | 7 (1.2) | 39 (7.0) |
| Hypertension | 191 (34.0) | 40 (7.1) | 231 (41.2) |
| Knee osteoarthritis | 35 (6.2) | 13 (2.3) | 48 (8.6) |
| MASH/MAFLD | 47 (8.4) | 23 (4.1) | 70 (12.5) |
| Musculoskeletal pain | 149 (26.6) | 50 (8.9) | 199 (35.5) |
| Obstructive sleep apnea | 27 (4.8) | 14 (2.5) | 41 (7.3) |
| PCOS | 13 (2.3) | < 6 | 14 (2.5) |
| Prediabetes | 9 (1.6) | < 6 | 10 (1.8) |
| Psoriasis | 35 (6.2) | 6 (1.1) | 41 (7.3) |
| T2D | 124 (22.1) | 35 (6.2) | 159 (28.3) |
| Urinary incontinence | 45 (8.0) | 13 (2.3) | 58 (10.3) |
| **CVD, any of**^a^ | 61 (10.9) | 23 (4.1) | 80 (14.3) |
| MI | 33 (5.9) | 8 (1.4) | 41 (7.3) |
| Unstable angina | < 6 | < 6 | 7 (1.2) |
| TIA | 8 (1.4) | < 6 | 13 (2.3) |
| Stroke | 24 (4.3) | 8 (1.4) | 32 (5.7) |
| **T2D microvascular complications, any of**^a^ | 34 (6.1) | 18 (3.2) | 50 (8.9) |
| Diabetic nephropathy | < 6 | – | < 6 |
| Diabetic retinopathy | 27 (4.8) | 8 (1.4) | 35 (6.2) |
| Diabetic neuropathy | < 6 | < 6 | < 6 |
| Diabetic foot | 7 (1.2) | 8 (1.4) | 15 (2.7) |

Abbreviations: CHF, chronic heart failure; CKD, chronic kidney disease; CVD, cardiovascular disease; GERD, gastroesophageal reflux disease; MASH/MAFLD, metabolic dysfunction-associated steatohepatitis/metabolic dysfunction-associated fatty liver disease; MI, myocardial infarction; ORC, obesity-related complication; PCOS, polycystic ovary syndrome; SIR, Salford Integrated Record; T2D, type 2 diabetes; TIA, transient ischemic attack.

^a^The ‘overall number of cases before and during follow-up’ may be smaller than the sum of cases ‘at or before index’ and ‘follow-up, incident’ because individuals with more than one complication/medical event would only be counted once.
